# Coronavirus misinformation and the political scenario: the science cannot be ‘another’ barrier

**DOI:** 10.1186/s13010-020-00092-5

**Published:** 2020-09-23

**Authors:** Marcelo Simões Mendes

**Affiliations:** Association for Psychological Science, Washington DC, United States of America

**Keywords:** Ideologies, Power, Health

## Abstract

The sensible and conflicting scenario of the pandemic postulated many challenges to societies around the world in 2020. Part of this problem refers to how the differences between politics and science are not comprehended in their particularities. The recognition of limits and power of science and politics can not only contribute to reaching the actions and strategies facing novel coronavirus but also optimized many domains of society.

## Background

The unprecedented consequences provoked by the novel coronavirus pandemic is not only situated on health field [1–3] but also all domains of society, such as economic [4], work [5], families [6], education [7], migration [8], and politics [9]. This complex scenario implies considering different levels of vulnerability [10], recruiting specific resources for singular needs.

In times of few certainties and many uncertainties, the precedence and quality of the knowledge can determine life or death. Especially from the field of uncertainties, different interests can conduce the social attitudes to subsequent terrible consequences.

In the political domain, the discussions crossed by multiple themes and different levels of understanding. From perspectives of conspiracy theories [11, 12] until politics of social distancing [13], most of the time, the politicians were judged and attacked whereas few times were defended.

## Politics and science: profound connection in different domains

The relationship of interdependence between politics and science provided a troubled disputed throughout history. On the coronavirus pandemic, this functioning was not different. However, Stevens [14] warns that total confidence in science at the level of determining politics means not understanding what science is.

A substantial reason that fosters this context of vulnerability is not to refer to false information, but how to own proprieties of some area of knowledge – including science and politics – are not understood in their conjectures. This comprehension implies recognizing the principles and limiting each area so as not to assume the same position as what it criticized (e.g., the historical debate between science and religion) [15].

The recent editorial published at Science, Thorp [16], argued the role of the antiscience movement to propagate misinformation and disinformation as a substantial barrier for advances in research on the novel coronavirus. Mainly the editorial focused on the relationship between misinformation and political scenario.

Despite the primordial importance of science contributing to reliable information on the contemporary scenario of ideological wars, another domain has interfered with the increase of misinformation and disinformation: the political polarization. The actions of political parties supported by correspondent interests from media have collaborated to strengthen these obstacles due to the development of false information and the elaboration of half-truths.

Extreme polarization on the contemporary political scenario has increased not just due to a pole or other but because of a profound relationship that supports then. There is a parallel relationship between the left and the right conjunctions in the political area. The alienated conceptions proliferate when the totality’s representations on the right identified in the opposite totality on the left – and vice versa.

This operation happens by logical implications: if something can exist just as itself or in its opposition, there are no possibilities for qualifying the singularities and pluralities. Thus, the right political position presents specific characteristics that differ from left political positions that have built throughout history.

Thus, the strengthening or maintenance of the political polarization potentiates a state of vulnerability in science and care practices. In a scenario that many spaces of knowledge are still ‘empty,’ half-true can be used as totally false or true, partially effective can be understood as totally effective or ineffective. Unfortunately, an unprecedented number of lives has paid for this battle.

## Conclusions

I disagreed with Thorp [16] when he argued that the only way to win this battle is to harness the same tools that are being used to bring down the science. This argument is the same mechanism that supports the political polarization and collaborates with misinformation and violence.

At least, the comprehension of science needs to be amplified because the delimitations of scientific knowledge depend on concepts, methods, and principles used. Another essential aspect to consider refers to the necessity to distinguish scientific knowledge and care practices. Both domains are different, and they need to be like that to qualify the experience of care. In summary, the best tool to win this battle is by science itself, with the recognition of its limits and its powerful reach.

## Data Availability

Not applicable.

## References

[1] Clark A, Jit M, Warren-Gash C, Guthrie B, Wang HHX, Mercer SW (2020). Global, regional, and national estimates of the population at increased risk of severe COVID-19 due to underlying health conditions in 2020: a modelling study. Lancet Glob Health.

[2] Javed B, Sarwer A, Soto EB, Mashwani ZR (2020). Impact of SARS-CoV-2 (coronavirus) pandemic on public mental health. Front Public Health.

[3] Giallonardo V, Sampogna G, Del Vecchio V, Luciano M, Albert U, Carmassi C (2020). Impact of quarantine and physical distancing following COVID-19 on mental health: study protocol of a multicentric Italian population trial. Front Psychiatry.

[4] Pak A, Adegboye OA, Adekunle AI, Rahman KM, McBryde ES, Eisen DP (2020). Economic consequences of the COVID-19 outbreak: the need for epidemic preparedness. Front Public Health.

[5] Coibion O, Gorodnichenko Y, Weber M. Labor markets during the Covid-19 crisis: a preliminary view. NBER. 2020.

[6] Prime H, Wade M, Browne DT (2020). Risk and resilience in family well-being during the COVID- 19 pandemic. Am Psychol.

[7] Colao A, Piscitelli P, Pulimeno M, Colazzo S, Miani A, Giannini S (2020). Rethinking the role of the school after COVID-19. Lancet Public Health.

[8] Chakraborty I, Maity P. COVID-19 outbreak: migration, effects on society, global environment and prevention. Sci Total Environ. 2020. 10.1016/j.scitotenv.2020.138882. 10.1016/j.scitotenv.2020.138882 PMC7175860 32335410

[9] McCloskey B, Zumla A, Ippolito G, Blumberg L, Arbon P, Cicero A (2020). Mass gathering events and reducing further global spread of COVID-19: a political and public health dilemma. Lancet..

[10] Boldt J (2020). The concept of vulnerability in medical ethics and philosophy. Philos Ethics Humanit Med.

[11] Douglas KM, Uscinski J, Sutton RM, Cichocka A, Nefes T, Ang J (2019). Understanding conspiracy theories. Polit Psychol.

[12] Douglas KM, Sutton RM, Cichocka A (2017). The psychology of conspiracy theories. Curr Dir Psychol Sci.

[13] Colbourn T. Unlocking UK COVID-19 policy. Lancet Public Health. 2020. 10.1016/S2468-2667(20)30135-3. 10.1016/S2468-2667(20)30135-3 PMC7266600 32502388

[14] Stevens A (2020). Governments cannot just ‘follow the science’ on COVID-19. Nat Hum Behav.

[15] Feyerabend P (1978). Science in a free society. Verso..

[16] Thorp HH (2020). Persuasive words are not enough. Science..

